# Epigenetic variation in early and late flowering plants of the rubber-producing Russian dandelion *Taraxacum koksaghyz* provides insights into the regulation of flowering time

**DOI:** 10.1038/s41598-024-54862-8

**Published:** 2024-02-21

**Authors:** Kai-Uwe Roelfs, Andrea Känel, Richard M. Twyman, Dirk Prüfer, Christian Schulze Gronover

**Affiliations:** 1 Fraunhofer Institute for Molecular Biology and Applied Ecology IME, 48149 Münster, Germany; 2 Institute of Plant Biology and Biotechnology, University of Münster, 48143 Münster, Germany; 3 TRM Ltd, Scarborough, UK

**Keywords:** Plant sciences, Plant development, Plant molecular biology

## Abstract

The Russian dandelion (*Taraxacum koksaghyz*) grows in temperate zones and produces large amounts of poly(*cis*-1,4-isoprene) in its roots, making it an attractive alternative source of natural rubber. Most *T. koksaghyz* plants require vernalization to trigger flower development, whereas early flowering varieties that have lost their vernalization dependence are more suitable for breeding and domestication. To provide insight into the regulation of flowering time in *T. koksaghyz*, we induced epigenetic variation by in vitro cultivation and applied epigenomic and transcriptomic analysis to the resulting early flowering plants and late flowering controls, allowing us to identify differences in methylation patterns and gene expression that correlated with flowering. This led to the identification of candidate genes homologous to vernalization and photoperiodism response genes in other plants, as well as epigenetic modifications that may contribute to the control of flower development. Some of the candidate genes were homologous to known floral regulators, including those that directly or indirectly regulate the major flowering control gene *FT*. Our atlas of genes can be used as a starting point to investigate mechanisms that control flowering time in *T. koksaghyz* in greater detail and to develop new breeding varieties that are more suited to domestication.

## Introduction

The Russian dandelion *Taraxacum koksaghyz* (2n = 2x = 16, family Asteraceae) is a promising alternative source of high-quality natural rubber and other secondary metabolites such as inulin and triterpenes^1–4^. Most natural rubber is currently sourced from the rubber tree (*Hevea brasiliensis*), which grows only in tropical regions and is restricted to certain soil types^5,6^. This species is cultivated in monocultures that are highly susceptible to pathogens, and its long generation time (lasting several years) means there is no rapid mechanism to introduce resistance traits and other beneficial characteristics^7^. The only way to increase yields is therefore to expand rubber tree plantations at the expense of rainforest^8^. In contrast, *T. koksaghyz* is a genetically amenable species with a short generation time (6–8 months) and the ability to grow in temperate zones and on nutrient-poor soils that do not support other crops^9,10^. However, the domestication of *T. koksaghyz* is hindered by further challenges such as self-incompatibility (which results in a high degree of heterozygosity) and the need for most plants to be exposed to prolonged cold (vernalization) to ensure homogeneous flowering in field stands, which is necessary for efficient seed harvesting within a short period of time.

The mechanisms that act before, during and after vernalization to regulate flowering have been studied in detail in the cruciferous model plant *Arabidopsis thaliana*. Before cold exposure, the transcription factor FLOWERING LOCUS C (FLC) acts as a central floral repressor, preventing the transcription of the major floral genes *FLOWERING LOCUS T* (*FT*) and *SUPPRESSOR OF OVEREXPRESSION OF CONSTANS 1* (*SOC1*)^11–13^. *FLC* expression is induced by interactions between multiple protein complexes that epigenetically establish an active chromatin environment at the *FLC* locus. One of these complexes is FRI-C, containing the floral regulator FRIGIDA^14^, which acts as a transcription factor by recruiting additional complexes such as COMPASS-like^15^, SWR1^16^ and PAF1^17^ to the *FLC* locus^18–20^. During prolonged cold, *FLC* expression is gradually suppressed as the chromatin structure changes in response to repressive histone markers such as the tri-methylation of K27 on histone H3 (H3K27me3), which is established by POLYCOMB REPRESSIVE COMPLEX 2^21,22^. *FLC* suppression also depends on VERNALIZATION INSENSITIVE 3 (VIN3)^21,23^, VIN3-LIKE 1 (VIL1)^24,25^, and the ubiquitination of histone H2A (H2Aub), which is established by POLYCOMB REPRESSIVE COMPLEX 1 and VIVIPAROUS1/ABI3-LIKE^26,27^. The suppression of *FLC* is still maintained when temperatures return to normal^28,29^. Under long-day conditions, the major circadian clock regulator GIGANTEA (GI) forms a complex with FLAVIN-BINDING, KELCH REPEAT, F-BOX 1 (FKF1) to mark the CONSTANS (CO) repressor CYCLING DOF FACTOR 1 for degradation. This allows CO to induce the expression of *FT* and subsequently *SOC1*, which promote flowering by inducing early floral meristem identity genes such as *LEAFY* (*LFY*) and *APETALA1*^30–35^. GI also acts independently of CO to promote the expression of miR172 and downregulate MADS-box protein SVP, AP2/ERF and the B3 domain-containing transcriptional repressor TEM1/2^36,37^. *FLC* expression is influenced not only by the photoperiodic, light quality and vernalization pathways, but also the autonomous pathway^38^ and the gibberellin pathway, which positively regulates *FT*, *SOC1* and *LFY*^39^.

Flowering is also induced by global DNA hypomethylation and repressed by specific localized hypermethylation, for example at the *FT* locus^40,41^. Whereas DNA methylation in mammals is largely restricted to the dinucleotide motif CpG, the trinucleotide motifs CHG and CHH are also methylated in plants (where H = A, T or C). The de novo methylation of plant DNA is mediated by the RNA-directed DNA methylation (RdDM) pathway involving DOMAINS REARRANGED METHYLASE 2 (DRM2) regardless of the sequence context^42,43^. However, maintenance methylation is motif-dependent, with CpG methylation maintained by METHYLTRANSFERASE 1 (MET1)^44^, CHG methylation by CHROMOMETHYLASE 2/3 (CMT2/3)^45,46^, and CHH methylation by CMT2 and DRM2^47^. Hypermethylation in promoter regions is often associated with gene silencing^48^, whereas the effects of downstream methylation are poorly understood^49^. Furthermore, the purpose of gene body methylation is contested, and may enhance splicing accuracy^50–52^, suppress gene expression^53^, induce gene expression^48,54^, suppress intragenic antisense transcripts^55^, or solely reflect the silencing of transposable elements (TEs)^56,57^, which are typically hypermethylated in all three contexts^48^.

In the family Asteraceae, the regulation of flower development has been studied in chrysanthemum^58^, gerbera^59,60^, and most recently the common dandelion *T. officinale*^61^, but the mechanisms of flower induction are poorly understood^62^. Furthermore, reproduction competence in perennials appears to be more complex than in annuals^63^. Flowering in *T. koksaghyz* can be induced 8 weeks after seeding (WAS) by a minimum of 2 weeks at 8 °C independent of a late flowering (LF) or early flowering (EF) phenotype^1–4^. LF plants need vernalization and were selected from populations that did not flower over a period of more than 6 months (spring to autumn) in the field. LF plants from different populations were then intercrossed several times to produce a stable vernalization dependent (VD) phenotype. To determine the factors that influence vernalization in *T. koksaghyz*, we used a reverse epigenetics approach by inducing changes in DNA methylation via the in vitro propagation of these VD plants representing different genotypes. The propagation of plants by tissue culture causes epigenetic reprogramming and somaclonal variation involving the loss of histone modifications and DNA methylation marks, and the reactivation of TEs^64–71^. Using this approach, we obtained *T. koksaghyz* plants with an EF phenotype from different genotype populations, indicating that the dependence on vernalization was abolished. We then applied whole-genome bisulfite sequencing (WGBS) to the EF plants and LF controls of each genotype to identify differentially methylated regions (DMRs) and associated genes.

As a second step to confirm that WGBS candidate gene expression was affected by vernalization, wild-type EF and LF plants grown in the field were intercrossed in two cycles followed by massive analysis of cDNA ends (MACE) to compare gene expression in leaf material before, during and after vernalization as well as reproductive (EF) and vegetative (LF) shoot apical meristem (SAM) samples. Our work provides insight into the control of flowering in *T. koksaghyz* and can be used as a starting point to test the function of candidate genes that may be involved in the regulation of flowering. A greater understanding of the control of flowering in *T. koksaghyz* will facilitate the breeding of vernalization-independent varieties with shorter life cycles and improved seed production, which can be used to meet the increasing demand for natural rubber.

## Results and discussion

### Induction of epimutations in *T. koksaghyz* plants

To study the impact of differential DNA methylation on flowering time in *T. koksaghyz*, we collected nine VD genotypes in the field and propagated ~ 40 individuals in vitro to induce changes in DNA methylation. After 26 weeks in the greenhouse, two EF plants (EF.TC) emerged for each of four genotypes, which were sampled together with three LF plants (LF.TC) of each genotype (Table S1). The EF plants were no longer dependent on vernalization. The abundance of secondary metabolites changes during plant development^72^, so we investigated the effect of the EF phenotype on the rubber content of root powder from all eight EF.TC and three LF.TC plants, but observed no significant differences in either the rubber content (Fig. S1, Table S2) or root biomass (Table S2). This contrasts with earlier rubber content studies, where comparisons were made between genotypes (rather than somaclonal epigenetic variants) or flowers were mechanically removed resulting in massive physiological changes in the plants^1^.

In apomictic *T. officinale*, stress-induced epigenetic changes are largely heritable^73^, so we assessed the stability of the EF phenotype by analyzing the recurrence of EF plants in the F_1_ generation. The *T. koksaghyz* genotypes that had yielded EF plants were therefore propagated again in vitro, together with one strictly VD plant as a crossing partner to overcome self-incompatibility. One of the genotypes produced six EF plants and another produced one EF plant after ~ 15 weeks. These were crossed with vernalized VD plants to produce an F_1_ population of 13 individuals, which were then monitored for the recurrence of the EF phenotype for 23 weeks. No further EF plants were observed. These results suggest that induced methylation changes can alter flowering behavior but the trait is not transmitted in a dominant manner. *T. koksaghyz* is thought to possess three loci with epistatic interactions affecting flowering habit^74^. The induced methylation changes may have affected one or more of these major loci, but factors with minor effects (not included in the three-locus model) may also have influenced the EF phenotype.

### Whole-genome bisulfite sequencing and analysis of DNA methylation patterns

**Figure 1:**
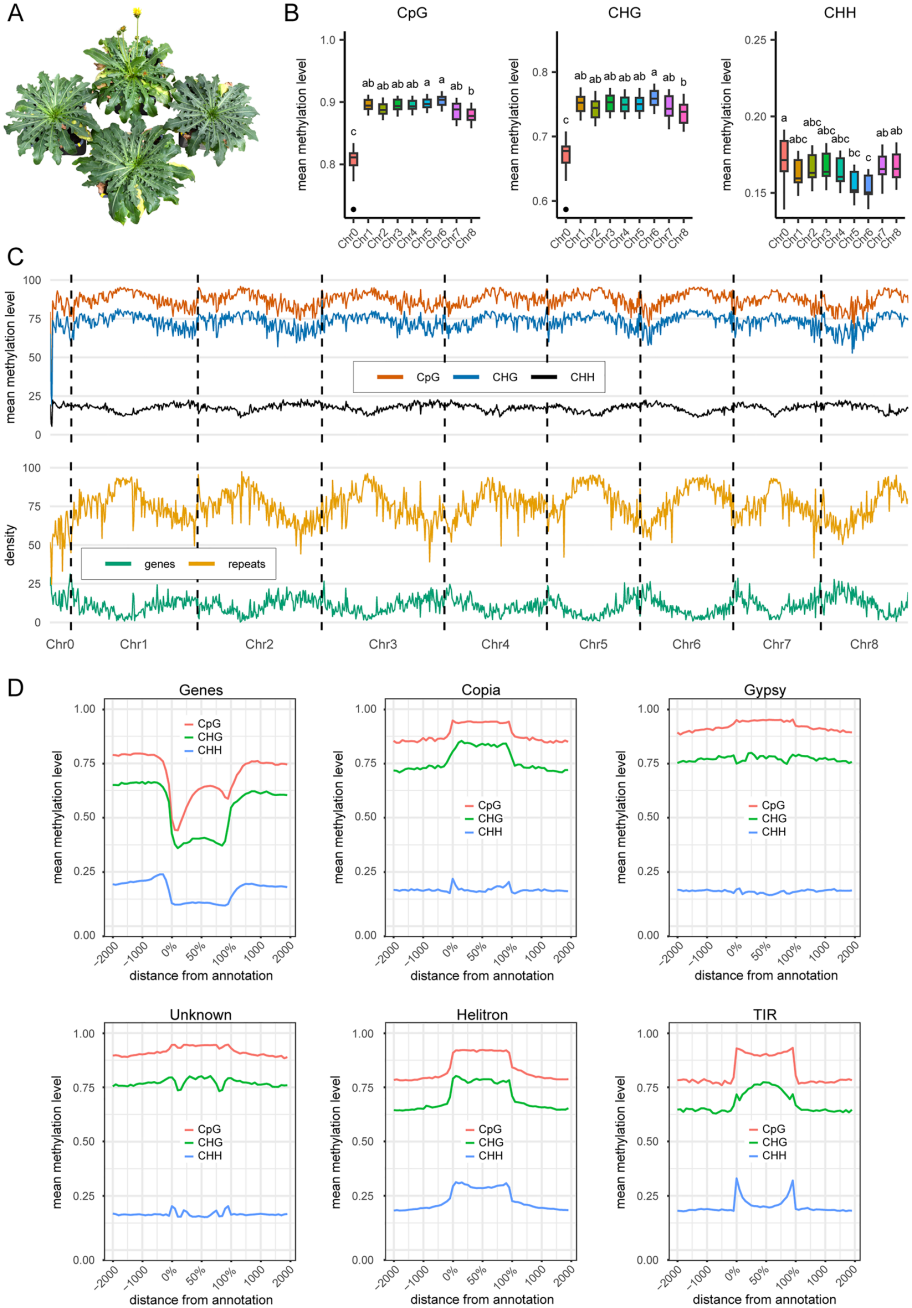
Plant genotypes and DNA methylation pattern analysis. (**A**) After in vitro propagation and the subsequent cultivation of nine different genotypes of vernalization-dependent *T. koksaghyz* plants in the greenhouse, clonal plants of four genotypes developed different flowering phenotypes – representatively shown for genotype 194/6–1, from which one EF and three LF plants were used for whole genome bisulfite sequencing (Table S1). (**B**) Global methylation levels per chromosome and context. Significant differences (*p* < 0.05; Tukey-HSD) are indicated by differing characters. (**C**) DNA methylation pattern at CpG, CHG and CHH sites and density of genes and repeats across all pseudochromosomes per megabase using a sliding window approach. (**D**) DNA methylation pattern at CpG (red), CHG (green) and CHH (blue) motifs in and 2 kb around predicted genes. LTR retrotransposons of type Copia, Gypsy and unknown, and DNA transposons of type Helitron and TIR-like are shown for sample Tarax-15 (LF). For all transposable elements except Helitron, only intact elements were investigated. EF = early flowering, LF = late flowering, TIR = terminal inverted repeat.

In order to identify DMRs potentially related to differences in flowering time between EF.TC and LF.TC plants, we applied WGBS to leaf material from one EF.TC and two or three LF.TC plants of each genotype (Fig. 1A, Table S1), resulting in four EF.TC and 11 LF.TC samples (Table S1). The EF.TC samples were acquired as soon as buds appeared. An average of ~ 436 million reads was generated for each sample, and 95.69–98.03% of the clean reads (~ 61.4 Gb of clean bases, representing > 56-fold coverage of the reference genome) remained after filtering for adapter sequences and low-quality bases (Table S3). Clean reads were mapped to the reference genome at an average rate of 95.56% excluding those failing quality control.

The methylation status of each covered cytosine residue in the three different contexts revealed a CpG mean methylation level (MML) of ~ 0.89 across all samples and pseudochromosomes, followed by CHG and CHH with scores of ~ 0.75 and ~ 0.16, respectively (Table S4). Genome-wide CpG and CHG methylation levels correlate with genome size, but this is not the case for CHH motifs^75^. With an estimated genome size of ~ 1.3 Gb^76^, the MML of *T. koksaghyz* is similar to that of sugar beet (*Beta vulgaris*) and, with the exception of CHH motifs, maize (*Zea mays*) even though the sugar beet genome is much smaller (714–758 Mb^77^) and the maize genome is much larger (~ 2.4 Gb^78^) than that of *T. koksaghyz*. Looking at each pseudochromosome individually (Fig. 1B), the MML for CpG and CHG motifs on Chr0 was significantly lower compared to the other pseudochromosomes, which is unsurprising because Chr0 only contains ambiguous sequences. The highest MML for CpG and CHG motifs was found on Chr6, but with a significant difference only compared to Chr8. Chr5 showed the same behavior, but only for the CpG motifs. However, Chr6 had the lowest MML for CHH motifs and differed significantly from Chr0, Chr7 and Chr8. Chr5 only differed significantly from Chr0.

We next compared the MML across entire chromosomes to the density of genes and TEs using a sliding window approach (Fig. 1C). TEs were identified by structure and homology, revealing several class I retrotransposons (Gypsy, Copia and unknown) as well as terminal inverted repeat (TIR)-like and Helitron class II DNA transposons representing up to 76.63% of the *T. koksaghyz* genome, in agreement with previous estimates^76^. Ignoring Chr0, the pattern of the other pseudochromosomes was clearly defined. CHH motifs were abundant at the terminal regions and scarcest in the middle, matching the distribution of genes, whereas CpG and CHG motifs were scarcest at the termini and more abundant in the middle, matching the distribution of TEs and other repeats. These findings indicate the most likely location of the pericentromeric region of each pseudochromosome, which is rich in heterochromatin, whereas euchromatin becomes increasingly abundant toward the distal regions, in line with DNA methylation patterns reported for *A. thaliana*^79^, rice (*Oryza sativa*)^54^ and maize^80^. However, CHH methylation tends to increase in the pericentromeric regions of all *A. thaliana* chromosomes.

We also assessed the specific MML pattern of all three motifs in the 2 kb flanking all genes and TEs (Fig. 1D). For genes, the abundance of methylated sites dropped immediately upstream of the transcription start site (TSS), but CHH motifs showed a slight increase in MML on the approach to the TSS. Furthermore, the MML for CHH motifs was minimal over the entire gene body, whereas methylated CpG (and to a lesser extent CHG) levels increased within the gene body to a maximum around the middle, but still lower than the level observed in the upstream and downstream regions, before decreasing slightly towards the transcription end site (TES). Finally, the abundance of methylation at all three motifs increased again just upstream of the TES, but the level was slightly lower than that just upstream of the TSS. This type of profile has been reported in several other plant species^75^, although the extent of gene body methylation varies greatly between species.

The opposite profile was observed for TEs, with the MML of CpG and CHG motifs increasing just upstream of the TE bodies. Copia, Helitron and TIR-like elements showed a steeper increase, whereas Gypsy and unknown elements were already hypermethylated in the upstream and downstream regions compared to the other elements. This increase persisted throughout the TE bodies and decreased to approximately the same level as in the upstream regions at the end of each TE. The CHG sites in Gypsy and unknown elements included a short region with minimal methylation just upstream of the end of each element. In contrast, CHH methylation did not persist throughout the TEs with the exception of the Helitron elements. The MML for CHH motifs in Gypsy elements was unremarkable, but in other TEs the level of methylation increased at the boundaries, particularly those of TIR elements. DNA methylation in all three contexts in *T. koksaghyz* therefore appears predominantly to silence TEs and prevent the often harmful effect of them spreading, as reported in other species^81^. However, not all TE types were methylated throughout at CHH sites. Most elements in genes were class II transposons^82,83^, but Copia-type elements were also found in genes^84,85^. This may also be related to the clear boundaries of CHH methylation in the TSS and TES of Helitron, TIR-like and (to a lesser extent) Copia-type elements, which may reflect a balance between active demethylation and de novo methylation by RdDM to prevent heterochromatin spreading into genic regions in *A. thaliana*^86,87^.

### Differential methylation and spatial proximity to genomic features

**Figure 2:**
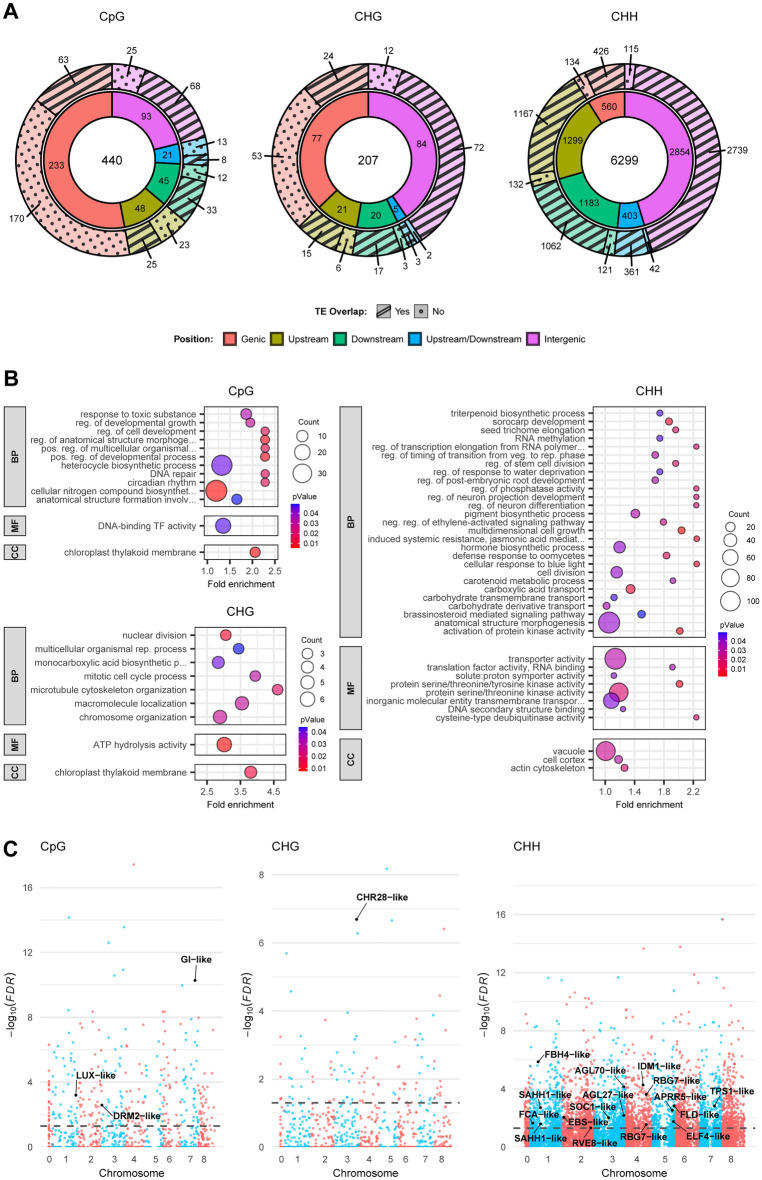
Differentially methylated regions (DMRs) and DMR-associated genes (DAGs) in EF.TC and LF.TC plants. (**A**) Position of DMRs in all three contexts relative to genes or their flanking regions (± 6 kb) as well as the fraction of overlap with transposable elements. (**B**) Gene Ontology enrichment analysis of DAGs in each methylation context. Fold enrichment refers to how much more frequently each term was observed than expected. BP = biological process, MF = molecular function, CC = cellular component. Significance level: *p * <  0.05 (weighted Fisher’s exact test). (**C**) Manhattan plot of DMRs for each context and chromosome. Chromosome boundaries are indicated by alternating colors. DMRs with candidate DAGs are labeled with gene symbols. The dashed bar denotes a significance level of FDR < 0.05 (Benjamini–Hochberg).

Next, we called DMRs independently for each context and mapped their spatial proximity to genes (± 6 kb) and TEs. We found 440, 207 and 6299 significant DMRs for the CpG, CHG and CHH contexts, respectively (Fig. 2A, Table S5).

The vast majority of DMRs were found in the CHH context, > 90% overlapping with TEs and most located in the intergenic space. CpG DMRs were the second largest group, and were predominantly found in genic regions. About a third of these also overlapped with TEs, but most of the TE overlap was proportionally found in intergenic and downstream regions. About 40% of CHG DMRs also overlapped with TEs in genic regions, and a similar proportion was found in intergenic regions, mostly overlapping with TEs.

A study of epigenetic changes during somatic embryogenesis in soybean (*Glycine max*) revealed an increase in DNA methylation in all three contexts (especially CHH sites) throughout the genome, strongly correlating with previously silenced TE-rich regions^88^. A genome-wide increase in DNA methylation in all three contexts was also observed in sugar beet callus compared to leaf tissue, where changes in CHH methylation levels were also predominantly found in repetitive sequences^89^. These findings coincide with the vast number of DMRs involving CHH motifs and their association with TEs in our study, which indicates that differences in methylation levels, arising during the differentiation of callus and plantlets propagated in vitro, are maintained through later stages of plant development.

TEs are often methylated in all three contexts (Fig. 1D), so it is unsurprising that most DMRs involving CHG and (to a lesser extent) CpG motifs are also associated with such regions. However, CpG motifs are predominantly found within gene bodies, and DMRs overlapping with TEs in upstream regions, and both upstream and downstream of genes, are not as overrepresented as those in intergenic and downstream regions, which may reflect the fact that CpG methylation also occurs in promoter regions and is associated with gene silencing.

Gene body methylation mostly affects CpG motifs and is prevalent in the coding region, whereas CHG methylation is often associated with TEs located in introns, as reported in *A. thaliana*^90^, soybean^91^ and maize^92^. This is consistent with the pattern of CpG and CHG methylation within gene bodies in *T. koksaghyz* (Fig. 1D). Nevertheless, several CpG DMRs were located in both gene bodies and TEs, whereas several CHG DMRs were found to overlap with gene bodies but not TEs. We identified 22 CpG DMRs that overlapped with introns for at least 50% of their length, only eight of which contained TEs, whereas only 10 CHG DMRs overlapped introns, six of which contained TEs. Moreover, several CHH DMRs were also located within gene bodies, but 285 overlapped with introns, of which 253 contained TEs. These findings indicate that methylation changes in gene bodies are induced by cultivation in vitro, not only in introns but also in coding regions. Non-CpG methylation is present in some genes and is associated with silencing^67,93,94^. However, some of these DMRs could be present in pseudogenes that are normally silenced, and those assigned to TEs may reflect the misclassification of genes that originate from TEs. Taken together, the differential methylation status of EF.TC and LF.TC plants mostly involves regions containing transposons, whereas gene body and promoter methylation differences are more likely to contribute to the change in phenotype.

### Gene Ontology and KEGG pathway enrichment analysis of DMR-associated genes

The DMR-associated genes (DAGs) in all three contexts were characterized by Gene Ontology (GO) (Fig. 2B) and KEGG pathway (Fig. S2) enrichment analysis relative to the entire *T. koksaghyz* gene pool.

CpG DAGs were enriched for GO terms including “circadian rhythm” (GO:0007623), “regulation of anatomical structure morphogenesis” (GO:0022603), “DNA-binding transcription factor activity” (GO:0003700) and “chloroplast thylakoid membrane” (GO:0009535). CHG DAGs were enriched for the GO terms “chromosome organization” (GO:0051276), “multicellular organismal reproductive process” (GO:0048609), and again “chloroplast thylakoid membrane”. Finally, CHH DAGs were enriched for the GO terms “cellular response to blue light” (GO:0071483), “regulation of stem cell division” (GO:2000035), “regulation of timing of transition from vegetative to reproductive phase” (GO:0048510), “carotenoid metabolic process” (GO:0016116), “brassinosteroid mediated signaling pathway” (GO:0009742) and “DNA secondary structure binding” (GO:0000217).

KEGG pathway enrichment analysis highlighted “carotenoid biosynthesis” (map00906), “MAPK signaling pathway—plant” (map04016), “mRNA surveillance pathway” (map03015) and “nucleotide excision repair” (map03420) for CHH DAGs. In contrast, the CpG and CHG DAGs were each enriched for “photosynthesis” (map00195), “linoleic acid metabolism” (map00591), “mismatch repair” (map03430) and “DNA replication” (map03030), among others.

These findings suggest that several genes involved in development, light signaling (including the circadian clock) and DNA metabolism may be affected by differential methylation, contributing to the EF phenotype.

### MACE analysis of leaf and SAM tissue from EF and LF plants

We complemented the epigenomic analysis of EF and LF plants with comparative transcriptomics to determine whether the DAGs are affected by vernalization and/or are differentially expressed during the transition to flowering. Accordingly, leaf material was harvested from EF plants shortly before the first floral primordia appeared (representing the time period of floral transition) and from LF plants before (LF.BV), during (LF.DV) and after vernalization (LF.AV). We also harvested SAM-enriched tissue during reproductive (EF.SR) and vegetative (LF.SV) growth. All samples were analyzed by MACE, generating an average of ~ 5.69 million reads (~ 0.38 Gb), ~ 5.2 million (~ 0.34 Gb, ~ 91.32%) of which could be mapped (Table S6). We identified differentially expressed genes (DEGs) by comparing the different leaf samples and the vegetative *vs* reproductive SAM tissues.

Hierarchical clustering of the 500 most variable genes revealed distinct clusters of gene expression either positively or negatively affected by vernalization, as well as substantial differences between the SAM and leaf samples in general (Fig. S3). All but one of the EF samples formed a tight cluster of replicates. The distance between the EF and LF.BV samples was small, indicating only subtle differences in gene expression. Interestingly, the LF.AV samples formed a separate cluster that was closer to the EF/LF.BV samples than the LF.DV samples, suggesting a strong effect on gene expression during vernalization followed by a return to pre-vernalization expression levels for many but not all genes. Furthermore, the vegetative and reproductive SAM samples formed separate clusters but the distance between them was small. These relationships were confirmed in a multidimensional scaling plot of DEGs showing that the 500 most variable genes were sufficient to separate each sample (Fig. S4).

We identified 5726 significant DEGs in at least one comparison from a total of 17,892 transcripts. They included 21 DEGs when comparing EF and LF.BV, 4177 between EF and LF.DV, 1389 between EF and LF.AV, 3173 between LF.BV and LF.DV, 625 between LF.BV and LF.AV, and 1673 between LF.DV and LF.AV. Finally, 40 DEGs were detected when comparing EF.SR and LF.SV.

### GO and KEGG pathway enrichment analysis of DEGs

GO and KEGG enrichment analysis of the DEGs (Figs. S5, S6) revealed no enrichment for any terms when comparing the EF and LF.BV samples, consistent with their similar expression profiles. However, we observed both similarities and subtle differences when we compared EF and LF.BV individually with the samples collected during vernalization (LF.DV). For example, both EF and LF.BV were enriched for terms associated with chloroplasts (GO:0009941, GO:0009570, GO:0009535) and response to cold (GO:0009409), but only LF.BV was enriched for the cellular responses to blue (GO:0071483), red (GO:0071491), far red (GO:0071490) and UV-A light (GO:0071492), and only EF was enriched for the category “photosynthesis, light harvesting in photosystem I” (GO:0009768). EF was also enriched for molecular function categories such as “chlorophyll binding” (GO:0016168) and “pigment binding” (GO:0031409). These partially overlapping but also partially unique enrichments were unsurprising because the radical change in temperature during vernalization differed greatly from the greenhouse conditions, affecting both the EF *vs* LF.DV and LF.BV *vs* LF.DV comparisons. In contrast, the change in response to light quality was small when comparing EF *vs* LF.DV but sufficient in the comparison LF.BV *vs* LF.DV to reveal differences in enrichment involving the response to various types of light, indicating a greater degree of similarity between EF and LF.DV than between LF.BV and LF.DV.

We also compared the EF and LF.BV samples individually with the samples collected after vernalization (LF.AV). The enrichment of EF samples for terms associated with chloroplasts persisted after vernalization, along with the addition of several further GO terms associated with the origin (GO:0005730), structure (GO:0022625, GO:0042788, GO:0022627, GO:0022626) and function (GO:0006412, GO:0002181, GO:0003735) of ribosomes. However, almost none of these terms were enriched when we compared the LF.BV and LF.AV samples, the exception being “plastid membrane” (GO:0005886). Instead, terms such as “DNA-binding transcription factor activity” (GO:0003700) and terms associated with the transport of auxins (GO:0080161), carboxylic acids (GO:0046942), amino acids (GO:0003333, GO:0015171) and inorganic molecular entities (GO:0015318) were overrepresented. This indicates subtle differences in the expression of chloroplast-related genes between the EF and LF.BV samples that were not apparent in the direct comparison.

When comparing samples acquired during and after vernalization (LF.DV *vs* LF.AV), all enriched terms associated with light quality (as discussed above) were enriched again, with the addition of “cellular response to high light intensity” (GO:0071486) as well as “response to cold”. This indicates that the expression profiles of the LF.AV and LF.BV samples are similar in this respect, as confirmed by their closer proximity in the heat map and multidimensional scaling plot (Figs. S3, S4).

Finally, DEGs in the comparison LF.SV *vs* EF.SR were enriched for the terms “floral meristem determinacy” (GO:0010582) and “specification of floral organ identity” (GO:0010093). This is unsurprising because these categories are directly related to the function of a reproductive SAM.

KEGG pathway analysis revealed the enrichment of “photosynthesis” (map00195) when comparing either LF.BV or EF with LF.DV, and when comparing LF.AV with EF, whereas “photosynthesis—antenna proteins” (map00196) and “MAPK signaling pathway—plant” (map04016) were also enriched when comparing either LF.BV or EF with LF.DV, and “ribosome” (map03010) was enriched when comparing LF.AV with EF. The “plant hormone signal transduction” pathway (map04075) was enriched when comparing either LF.BV or LF.AV with LF.DV, and the latter comparison was also enriched for “circadian rhythm—plant” (map04712).

Taken together, the differences between EF and LF.BV appear mostly to reflect the expression of genes involved in pathways associated with light quality responses and processes localized in plastids, largely matching the enriched terms and pathways associated with the DAGs.

### Candidate genes for flowering time regulation

We screened the most significant DAGs to identify candidate genes that are likely to encode regulators of flowering time in *T. koksaghyz* based on their homology to functionally relevant genes in *A. thaliana*. However, we evaluated them in more closely related plants because there are likely to be functional differences between the families Brassicaceae and Asteraceae^62,95,96^. We also investigated genes that may affect chromatin structure, and whose dysregulation may therefore contribute to the EF phenotype. Finally, we compared the DEGs derived from MACE analysis to all the significant DAGs in order to identify intersections and further evidence of regulation (Fig. S7).

**Figure 3:**
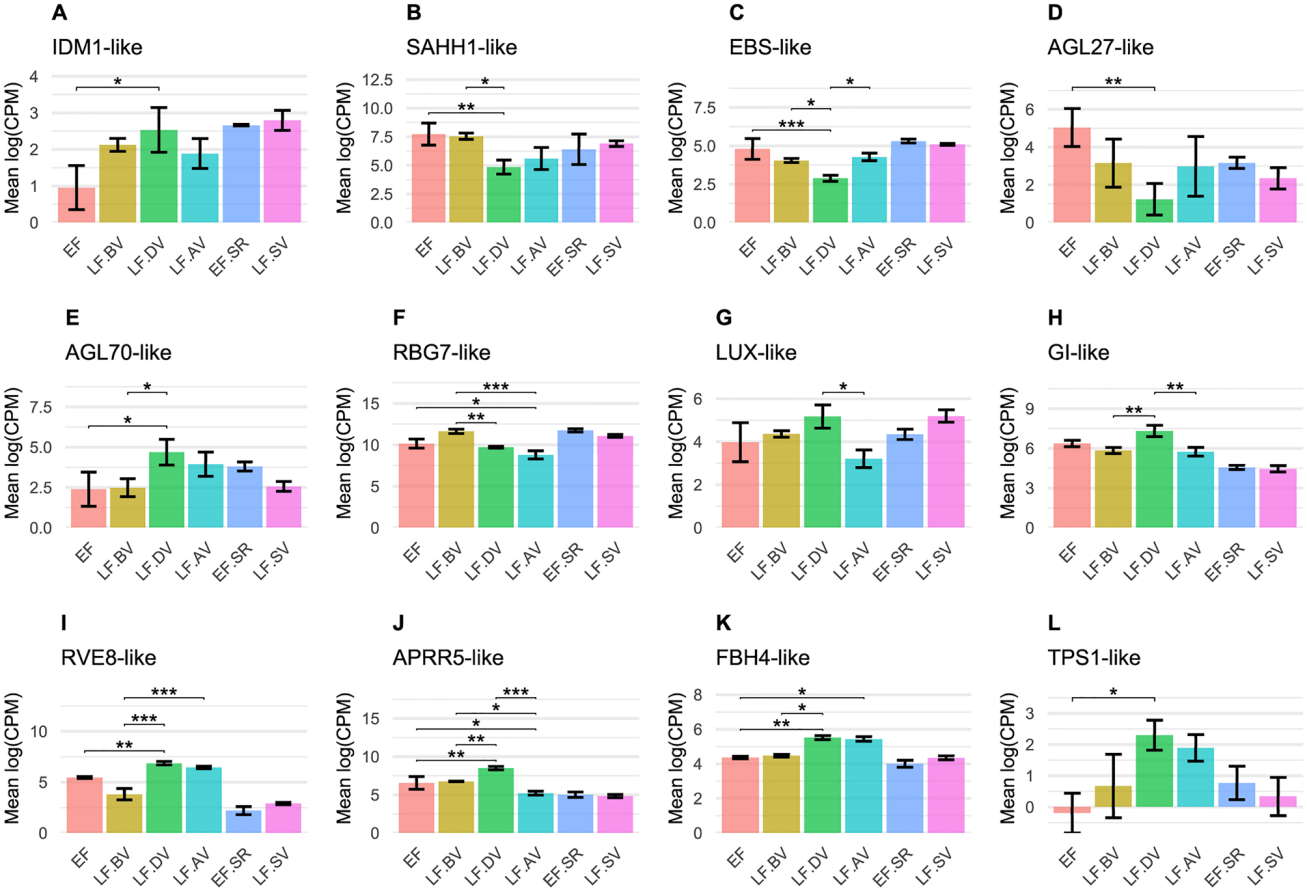
Expression levels of differentially expressed candidate DMR-associated genes (DAGs) in early and late flowering plants before, during and after vernalization. Expression levels of 12 candidate genes (**A**–**L**) were analyzed by MACE using leaf tissue (EF, LF.BV, LF.DV, LF.AV) or shoot apical meristem-enriched tissue (EF.SR, LF.VR). Data are means ± SD, *n* = 3 biological replicates (EF, LF.BV, LF.DV, LF.AV), *n* = 3 pools of five biological replicates (EF.SR, LF.VR); * = FDR < 0.05, ** = FDR < 0.005, *** = FDR < 0.0005 (Benjamini-Hochberg). DMR = differentially-methylated region, EF = early flowering, LF = late flowering, BV = before vernalization, DV = during vernalization, AV = after vernalization, SR = shoot apical meristem (reproductive), SV = shoot apical meristem (vegetative).

We found that 639 of the 5726 DEGs were also defined as DAGs. The greatest overlap was found for CHH DAGs (567, 15.14%), followed by CpG (49, 13.17%) and CHG DAGs (8, 5.97%), and then DAGs with combined CpG/CHG (6), CpG/CHH (6) and CHG/CHH (3) motifs. There were no overlaps between the DEGs and DAGs containing mixtures of all three motifs. A selection of candidate DAGs was characterized in more detail (Table 1; Fig. 2C), and those with differential expression in at least one comparison are shown in Fig. 3. Other interesting candidate DAGs with or without supporting MACE data are listed in Table S7.

**Table 1:** Candidate genes associated with differentially methylated regions.

Accession	Mean methylation difference^a^	Context	Location	UniProt accession^b^	Percent identity^b^	Annotation^b^	Gene symbol	Category	Function in *A. thaliana*	References
TkA03G010630	− 0.47	CpG	Exon 8	Q9M548	54.46	Protein DOMAINS REARRANGED METHYLASE 2	*DRM2-like*	DNA methylation and histone modification	Major DNA methyltransferase responsible for de novo DNA methylation in the RdDM pathway	^42,47^
TkA03G561890	− 0.75	CHG	Exon 4	Q94BR5	54.58	Protein CHROMATIN REMODELING 28	*CHR28-like*	DNA methylation and histone modification	Nucleosome repositioning; associates with SUVR2; required for methylation of several RdDM target loci	^127,128^
TkA04G343790	0.33	CHH	Upstream	F4IXE7	53.89	Increased DNA methylation 1	*IDM1-like*	DNA methylation and histone modification	Histone H3 acetyltransferase acting on loci possessing DNA methylation but lacking H3K4me2/me3	^129,130^
TkA01G206370	− 0.35	CHH	Upstream	O23255	93.20	Adenosylhomocysteinase 1	*SAHH1-like*	DNA methylation and histone modification	Hydrolysis of S-adenosyl-homocysteine formed as a by-product of and competitively inhibiting methyltransferase reactions	^131,132^
	0.4									
TkA02G027650	0.35	CHH	Upstream	F4JL28	78.80	Protein EARLY BOLTING IN SHORT DAYS	*EBS-like*	DNA methylation and histone modification	Binding and prevention of acetylation of methylated histone H3 through the recruitment of HDA6	^133^
TkA03G581150	0.36	CHH	Upstream	Q9AT76	31.69	Agamous-like MADS-box protein AGL27	*AGL27-like*	Vernalization and autonomous	Repressor of FT; partly required for FLC-dependent flowering repression; forms nuclear complexes with other members of its clade; interacts with SVP	^101,134,135^
TkA03G581530	0.5	CHH	Intron 8	Q9LSR7	41.76	Agamous-like MADS-box protein AGL70	*AGL70-like*	Vernalization and autonomous	Repressor of FT; partly required for FLC-dependent flowering repression; forms nuclear complexes with other members of its clade	^101,135^
TkA04G406810	− 0.39	CHH	Upstream	Q03250	83.53	Glycine-rich RNA-binding protein 7	*RBG7-like*	Vernalization and autonomous	Involved in alternative splicing of various target mRNAs; promotes flowering independent of photoperiodic and vernalization pathways	^136,137^
	− 0.27									
TkA01G050830	0.43	CHH	Upstream	O04425	61.54	Flowering time control protein FCA	*FCA-like*	Vernalization and autonomous	Repression of sense and antisense FLC transcripts	^138–140^
TkA05G444010	− 0.34	CHH	Upstream	Q9CAE3	77.12	Protein FLOWERING LOCUS D	*FLD-like*	Vernalization and autonomous	Histone demethylase involved in the repression of FLC; promotes flowering independent of photoperiodic and vernalization pathways; interacts with FCA	^139,141,142^
TkA01G602290	0.46	CpG	Exon 1	Q9LTH4	45.76	Protein LUX ARRHYTHMO	*LUX-like*	Photoperiodism and circadian clock	Transcription factor and DNA-binding component of the evening complex; binds to the evening element of other circadian clock genes like CCA1 and LHY	^106,143,144^
TkA05G430470	− 0.42	CHH	Downstream	O04211	56.04	Protein EARLY FLOWERING 4	*ELF4-like*	Photoperiodism and circadian clock	Member of the evening complex involved in circadian clock function	^106,145^
TkA07G372490	− 0.5	CpG	Exon 8	Q9SQI2	70.14	Protein GIGANTEA	*GI-like*	Photoperiodism and circadian clock	Involved in the circadian clock; positive regulator of flowering in a CO-dependent and CO-independent manner	^34,36,37,146^
TkA02G547190	0.39	CHH	Downstream	Q8RWU3	67.98	Protein REVEILLE 8	*RVE8-like*	Photoperiodism and circadian clock	Transcription factor involved in the circadian clock by positively regulating TOC1 via the promotion of histone acetylation	^147,148^
TkA05G405710	− 0.41	CHH	Upstream	Q6LA42	40.66	Two-component response regulator-like APRR5	*APRR5-like*	Photoperiodism and circadian clock	Transcriptional repressor of the circadian clock components CCA1 and LHY	^149^
TkA01G157760	0.56	CHH	Upstream	Q66GR3	37.56	Transcription factor bHLH130	*FBH4-like*	Photoperiodism and circadian clock	Promotion of flowering by positively regulating CO	^150^
TkA07G297150	− 0.3	CHH	Intron 3	Q9SYM4	80.18	Trehalose-6-phosphate synthase 1	*TPS1-like*	Photoperiodism and circadian clock	Integration of high carbohydrate level signals into the flowering pathway to promote flowering upstream of FT	^151,152^
TkA03G288970	0.32	CHH	Upstream	O64645	70.86	Protein SUPPRESSOR OF CONSTANS OVEREXPRESSION 1	*SOC1-like*	Floral integrator	Transcription factor integrating multiple flowering signals to promote flowering	^153,154^

^a^EF.TC relative to LF.TC.

^b^Based on a BlastX search against the UniProt SwissProt database.

In terms of factors likely to be involved in the modulation of DNA methylation patterns, one particular gene with homology to *A. thaliana DRM2* stood out due to the strong CpG hypomethylation of exon 8 in the EF.TC plants (Table 1). The corresponding sequence is part of exon 9 in the *A. thaliana DRM2* gene, and is normally subject to heavy CpG methylation^97,98^. It is unclear whether this hypomethylation influences the expression or splicing of the *T. koksaghyz DRM2-like* gene, but it may, at least in part, be responsible for the DNA methylation differences in both phenotypes (especially in the CHH context). Interestingly, we identified another key component of RdDM, a homolog of CHROMATIN REMODELING 28 (CHR28/FRG2) (Table 1). The *T. koksaghyz CHR28-like* gene was hypomethylated in exon 4, which may have influenced its expression and thus contributed to the EF phenotype by suppressing the activity of RdDM. A *T. koksaghyz* homolog of the histone H3 acetyltransferase INCREASED DNA METHYLATION 1 (IDM1) was also identified (Table 1). The expression level of the *T. koksaghyz IDM1-like* gene was significantly lower in EF than LF.DV plants (Fig. 3A). Because hypermethylated promoters often correlate with gene silencing, this finding suggests *IDM1-like* has a key role (probably involving an unidentified ROS1 homolog) in the de-repression of flowering time control genes, and the dysregulation of *IDM1-like* caused by promoter hypermethylation may have triggered the hypermethylation of other loci in the EF.TC plants. A homolog of *ADENOSYLHOMOCYSTEINASE 1* (*SAHH1*) was found to contain two DMRs, one closer to the TSS hypomethylated region and the other hypermethylated and about 4 kb upstream (Table 1). The expression of *SAHH1-like* was inhibited by vernalization, which probably suppressed methyltransferase reactions due to an excess of S-adenosyl-homocysteine (Fig. 3B). Several genes involved in DNA methylation therefore appear to be differentially methylated in EF.TC *vs* LF.TC plants after propagation in vitro, and some also seem to respond to vernalization.

We also considered components of the vernalization and autonomous pathways. For example, we identified a homolog of *EARLY BOLTING IN SHORT DAYS* (*EBS*)^99,100^ that was hypermethylated in its upstream region (Table 1). This *EBS-like* gene was significantly downregulated during vernalization compared to the periods before and after (Fig. 3C). We identified the FLC homologs^101–104^ AGL27 (also known as MAF1/FLM) and AGL70 (also known as MAF3), which are therefore likely to be floral repressors (Table 1). *AGL27-like* was hypermethylated in its upstream region and *AGL70-like* within intron 8. Interestingly, *AGL27-like* was downregulated during vernalization whereas *AGL70-like* showed the opposite profile (Fig. 3D,E). We also identified a homolog of GLYCINE-RICH RNA-BINDING PROTEIN 7 (RBG7/GRP7) (Table 1), which promotes the repressive splicing variant AGL27-β as well as H3K4 demethylation at the *FLC* locus, thus inhibiting *FLC* expression when overexpressed in *A. thaliana*^105^. *RBG7-like* contained two upstream hypomethylated sequences in the EF.TC plants, and was expressed at significantly lower levels during vernalization (compared to LF.BV but not EF) and even more so afterwards (Fig. 3F). Furthermore, we identified homologs of the FLC repressors *FLOWERING TIME CONTROL PROTEIN FCA* (*FCA*) and *FLOWERING LOCUS D* (*FLD*), the first being hypermethylated and the second hypomethylated in their upstream regions (Table 1). The corresponding proteins are thought to cooperate with RBG7^105^. The functional analysis of *AGL27-like*, *AGL70-like*, *FCA-like* and *FLD-like* should be prioritized because an *FLC-like* gene has yet to be identified in *T. koksaghyz*.

Looking at candidate genes involved in the photoperiodic pathway and circadian clock, we identified homologs of LUX and ELF4, two components of the evening complex (Table 1). *ELF4-like* was hypomethylated in its downstream region, whereas *LUX-like* was hypermethylated in its only exon. *LUX-like* was also slightly upregulated during vernalization and significantly downregulated afterwards (Fig. 3G). The slight upregulation of *LUX-like* during vernalization suggests a role during flowering, but it is unclear whether *ELF4-like* interacts with *LUX-like* in *T.* *koksaghyz* to form an evening complex similar to that found in *A. thaliana*^106^. We found a *T. koksaghyz* homolog of *GI* with strong hypomethylation in exon 8 in the EF.TC plants, which was significantly upregulated during vernalization compared to the periods before and after (Fig. 3H). Homologs of the circadian clock components RVE8 and APRR5 were also identified (Table 1). *RVE8-like* was hypermethylated in its downstream region in EF.TC plants and it was expressed at a slightly higher level in EF *vs* LF.BV plants but was strongly induced during and after vernalization (Fig. 3I). *APRR5-like* was strongly hypomethylated in its upstream region and was similarly expressed in EF and LF.BV plants but significantly upregulated during vernalization followed by a sharp decline afterwards (Fig. 3J). *APPR5-like* in *T. koksaghyz* may play a similar role to sugar beet BTC1 in the control of flowering time, but the presence of a negative feedback loop between *RVE8-like* and *APRR5-like* (as proposed for *A. thaliana*) and their interactions with yet-to-be-identified FT homologs require further investigation. Two further photoperiodic candidate genes were also identified: *FBH4-like* was hypermethylated in its upstream region in EF.TC plants, whereas *TPS1-like* was hypomethylated within intron 3. *FBH4-like* was significantly upregulated during and after vernalization, suggesting it promotes flowering (Fig. 3K), whereas *TPS1-like* was expressed at minimal levels in EF and LF.BV plants followed by a sharp increase during vernalization and consistent high levels thereafter (Fig. 3L). The upregulation of *TPS1-like* in *T. koksaghyz* may be a response to cold treatment and its putative role in the regulation of flowering time needs further investigation.

## Conclusions

Our combination of epigenomic and transcriptomic analysis provided a list of candidate genes characterized by DMRs and/or differential expression patterns that correlated with vernalization and the timing of flower development, thus offering insight into the regulation of flowering time in *T. koksaghyz* and its potential utilization in the domestication of this species. The comparison of early flowering plants and late flowering controls allowed us to identify differences in methylation patterns (particularly genic CpG methylation sites) and expression profiles affecting genes associated with vernalization and photoperiodism. The most promising candidate genes were those directly involved in epigenetic modification, and those encoding components of the vernalization and autonomous pathways, the photoperiodic pathway and circadian clock. We refined a list of candidate flowering time control genes by incorporating WGBS and MACE datasets from EF and LF *T. koksaghyz* plants*.* This atlas of genes for the multiparametric and quantitative trait of flower development can be used for an initial investigation of the regulatory mechanisms that control flowering time in *T. koksaghyz*.

## Methods

### Plant material and growth conditions for WGBS

Nine individual *T. koksaghyz* plants from a wild-type population of half-siblings were selected based on the absence of flowering after 26 weeks growing in the field. The plants, provided by ESKUSA (Straubing, Germany), were then propagated by sterile culture in vitro to produce 20–40 individuals as previously described^107^ but without antibiotics for selection. These individuals were cultivated in the greenhouse at 14–18 °C (night) and 22–25 °C (day) with a 16 h photoperiod. The light intensity was 30 klx provided by Phillips 400-W SON-T Agro NDL high-pressure sodium lamps. The plants were grown in TKS1 cultivation substrate (Floragard, Oldenburg, Germany) and were monitored for up to 26 weeks. When a flowering plant appeared, leaf material from that plant and three non-flowering plants of the same genotype was frozen in liquid nitrogen. The samples are described in detail in Table S1.

### Analysis of natural rubber content

Roots were collected from individual LF.TC and EF.TC plants after 26 weeks and were dried at 40 °C for 2 weeks before grinding to powder. We then determined the poly(*cis*-1,4-isoprene) content of 50 mg samples by proton nuclear magnetic resonance (^1^H-NMR) spectroscopy as previously described^108^.

### Library preparation and WGBS

WGBS libraries were prepared from leaf material by AllGenetics & Biology (Oleiros, Spain) and sequenced on their HiSeq XTen PE150 platform to generate 2 × 150-bp reads with 50 × coverage.

### WGBS data analysis

Raw reads were quality controlled and adapters were removed using BBDuk in BBTools suite v38.92^109^. Clean reads were mapped to the *T. koksaghyz* reference genome^76^ using bwa-meth v0.2.5^110^. TEs in the reference genome were predicted using EDTA^111^. DNA methylation patterns were extracted using MethylDackel v0.6.0 (https://github.com/dpryan79/MethylDackel/) with a minimum depth of four supporting reads and a MAPQ > 10 cutoff. Cytosine positions with > 300 × coverage were manually excluded. DNA methylation patterns were analyzed using methylKit v1.24.0^112^ and METHimpute v1.20.0^113^ in R v4.2.1. Replicates Tarax-5 and Tarax-8 were excluded from DMR analysis based on principal component analysis of all cytosine residues (Fig. S8) and context-specific DMRs were called with at least two EF and six LF replicates using metilene v0.2-8^114^. The DMRs were filtered by imposing a false discovery rate (FDR) threshold < 0.05. The DNA methylation pattern and density of genes and repeats per pseudochromosome were determined using a sliding-window approach (1 Mb) with a custom *Python* script. DMRs near genes (up to 6 kb either side) were identified using the *intersect* function of BEDtools v2.30.0^115^. Briefly, each DMR was tested for complete overlap with a gene plus downstream and upstream extensions, then classified by its exact location relative to candidate genes. DMRs were considered to be in genic regions or TEs (score ≥ 300) if they overlapped by at least 1 bp. Genes were annotated against the NCBI RefSeq^116^ (download date 22.09.22) and UniProt SwissProt^117^ (download date 17.08.22) databases using *blastx* in Blast + Suite v2.13.0 + ^118^. GO enrichment analysis was carried out using the topGO R package v2.48.0^119^ with the *weight_fisher* algorithm and a significance level of *p* < 0.05. KEGG pathway enrichment analysis was carried out using the *enricher* method in the clusterProfiler R package v4.4.4^120^ with a significance level of *p* < 0.05. GO and KEGG pathway data were retrieved using the eggNOG-Mapper online tool^121^ (access date 12.04.2023).

### Plant growth conditions for assessment of phenotypic stability

The *T. koksaghyz* plants used for WGBS were propagated in vitro as above to produce 15–17 further individuals. Half-siblings cannot be crossed effectively, so one individual from a mixed population of strictly VD plants was also propagated in vitro to produce six individuals. The WGBS plants were transferred to soil 3 weeks after the VD plants, and both were grown under the conditions described above. In addition, after 8 weeks of cultivation, the VD plants were vernalized in a Percival LT-36VL phytochamber (CLF Plant Climatics, Wertingen, Germany) at 4 °C and 22 klx light intensity with an 8-h photoperiod for 3 weeks and then returned to the greenhouse. Flowering plants from both experiments were crossed and the harvested seeds were germinated under the same conditions as above. The resulting plants were monitored regularly for ~ 15 weeks to check for the re-occurrence of flowering.

### Plant material and growth conditions for MACE

We selected 12 EF and 18 LF *T. koksaghyz* plants from a wild-type mixed population of six and eight crossing families. Both groups were internally crossed, and seeds from five mother plants each were sown in separate batches to generate F_1_ plants. The seedlings were initially grown in VM propagation substrate and then transferred to ED73 standard soil (both from Einheitserde, Sinntal-Altengronau, Germany). The plants were cultivated in the greenhouse with the same temperature and photoperiod conditions as described above, supported by 600 W high pressure sodium lamps (Greenbud, Wischhafen, Germany). When 84–93% of the EF F_1_ plants spontaneously flowered within 13 WAS, crosses were carried out to generate F_2_ progeny. Conversely, when 81–92% of the LF F_1_-plants did not flower within 22 WAS, the non-flowering plants were vernalized in a vernalization chamber as described above. After 3 weeks, the plants were transferred back to the greenhouse for crossing and seed harvest. For each phenotype, we grew the F_2_ progeny of three specific crosses. For MACE, leaf material was harvested individually from 102 EF plants and 107 LF plants at 6 WAS (LF.BV). At 7 WAS, all plants were phenotyped for the emergence or absence of floral primordia, and 36 EF and 30 LF plants were selected for harvesting reproductive (EF.SR) and vegetative (LF.SV) SAM-enriched tissue. At 13 WAS, 42 LF plants were vernalized as described above, and at 14 WAS, leaf material was sampled from plants during vernalization (LF.DV). The plants were then transferred back into the greenhouse at 16 WAS and leaf material was collected from each plant at 17 WAS (LF.AV). Samples were always collected at the same time point in the afternoon and each sample was snap-frozen in liquid nitrogen.

### RNA extraction for MACE

We prepared three biological replicates of leaf material for each of the four time points and three pools of about five samples for the vegetative and reproductive SAM-enriched tissues. The samples were ground to powder under liquid nitrogen using a mortar and pestle and total RNA was isolated using the innuPREP Plant RNA Kit (Analytik Jena, Jena, Germany). The RNA quality was determined by spectrophotometry and agarose gel electrophoresis.

### Library preparation and MACE

MACE sample processing, quality control and sequencing were carried out by GenXPro (Frankfurt am Main, Germany) using their standard protocol for the HiSeq 4000 and NextSeq 500 platforms to produce 5–10 × 10^6^ raw 1 × 75 bp reads per sample. Briefly, polyadenylated and fragmented RNA was transcribed into cDNA using specific oligonucleotides, one of which included a barcode (TrueQuant Technology) for PCR bias elimination, allowing each mRNA molecule to be represented by a single read.

### MACE data analysis

Reads were mapped to the *T. koksaghyz* reference genome^76^ and quantified using subread-align (at most five best-quality alignments reported) and featureCounts (multi-mapping reads counted fractionally) in Subread v2.0.3^122^. Raw counts were examined using edgeR v3.38.4^123^ for differential expression analysis and multidimensional scaling. We calculated p-values and FDRs using a minimum of log_2_(1.5). For each comparison, genes were considered differentially expressed at an FDR threshold < 0.05. GO and KEGG pathway analysis were carried out using the topGO (*weight_fisher* algorithm, FDR < 0.1) and clusterProfiler R packages (FDR < 0.1), respectively. The heat map was created from the 500 most variable genes based on counts per million using the heatmap.2 function of gplots R package v3.1.3^124^. The box plot and bar plot were created using the ggplot2 R package v3.4.2^125^.

### Comparative analysis

Venn diagrams were generated using the online tool *InteractiVenn*^126^, accommodating all genes associated with significant DMRs as well as those that were significantly differentially expressed.

### Statement on experimental research and field studies on plants

The field-cultivated plant material used in this study was provided by ESKUSA GmbH, Germany. All methods were carried out in accordance with relevant guidelines for the collection of plant material and relevant institutional, national and international guidelines and legislation.

### Supplementary Information

Supplementary Tables.

Supplementary Figures.

## Data Availability

Sequence data are available at the NCBI sequence read archive (accession no. PRJNA1066591).
