# Supplementary material for: Epigenetic variation in early and late flowering plants of the rubber-producing Russian dandelion Taraxacum koksaghyz provides insights into the regulation of flowering time
Source: Sci Rep. 2024 Feb 21;14:4283. doi: 10.1038/s41598-024-54862-8 (PMC10881582; doi:10.1038/s41598-024-54862-8)
Supplement: Supplementary file 2 — Supplementary Figures. [file 41598_2024_54862_MOESM2_ESM.docx]

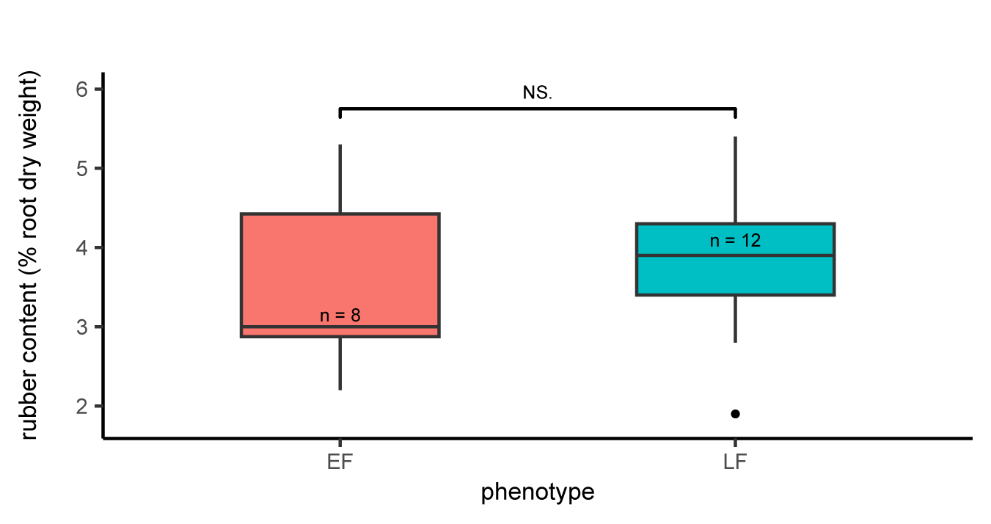


**Figure S1:** Rubber content of early and late flowering *T. koksaghyz* plants used for whole genome bisulfite sequencing.

Roots were collected from individual LF.TC and EF.TC plants after 26 weeks and were dried at 40 °C for 2 weeks before grinding to powder. The rubber content in early flowering (EF) and late (LF) flowering plants was measured as the poly(cis-1,4-isoprene) content of root powder by ^1^H-NMR spectroscopy. In the boxplots, center line = median, box limits = upper and lower quartiles, and whiskers = 1.5× interquartile range; *n* = 8 (EF), *n* = 12 (LF). Significance level: p < 0.05 (Wilcoxon); NS = not significant.


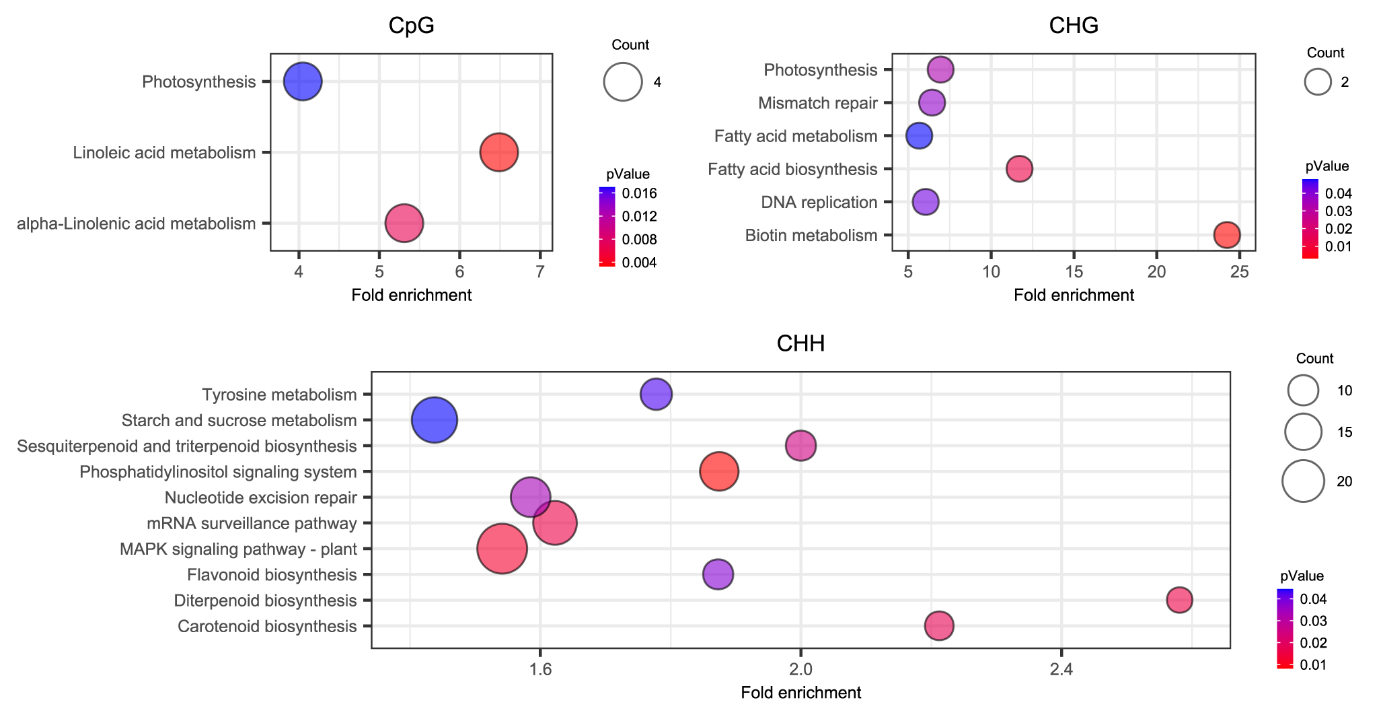


**Figure S2:** KEGG pathway enrichment analysis of DAGs in each methylation context

Fold enrichment refers to how much more frequently each term was observed than expected. Significance level: p < 0.05 (hypergeometric test).


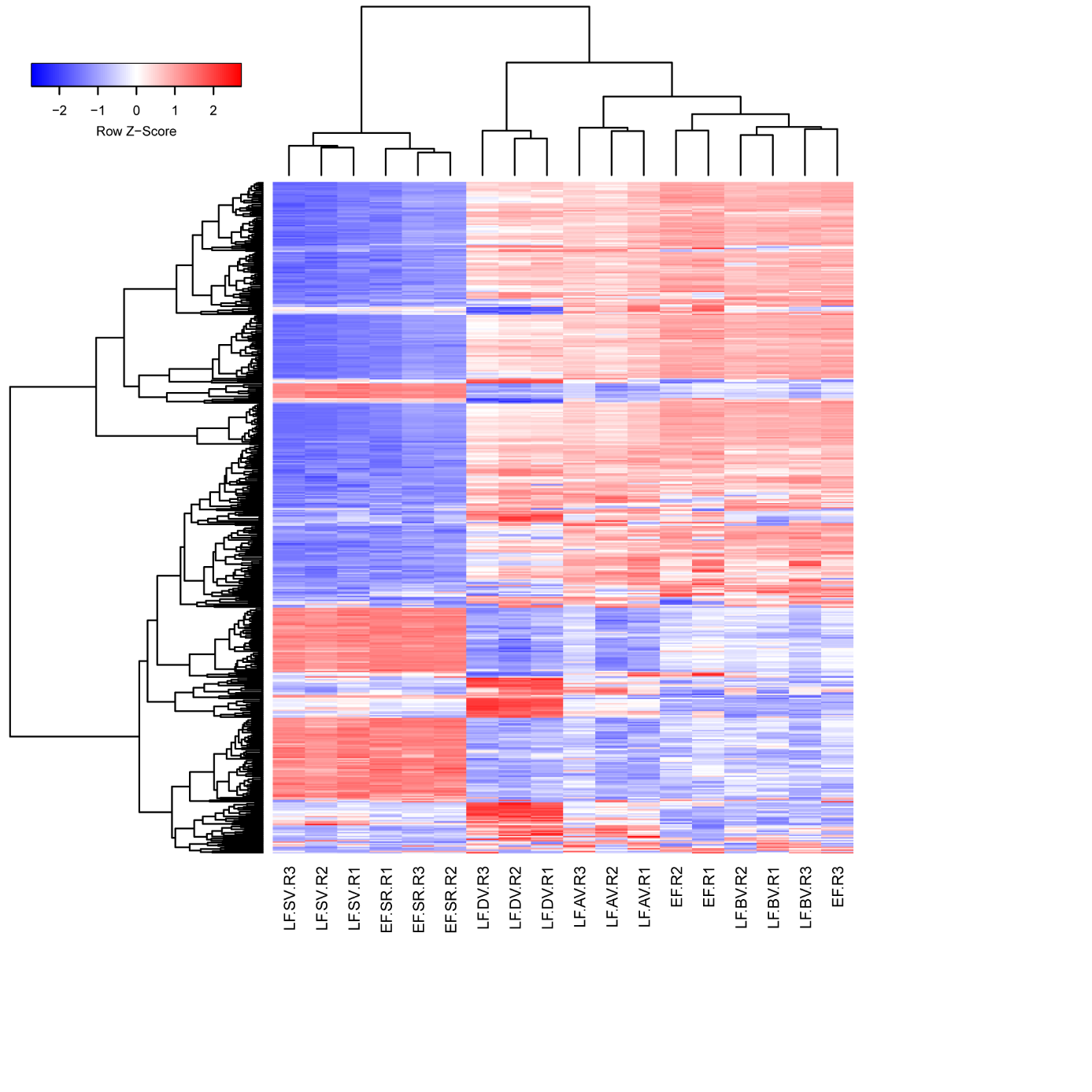
**Figure S3:** Heat map with dendrograms showing hierarchical clustering of the 500 most variable genes detected by MACE in early flowering and late flowering plants before, during and after vernalization.

The heat map shows individual replicates (1–3) for each sample. Red indicates upregulation and blue indicates downregulation. EF = early flowering, LF = late flowering, BV = before vernalization, DV = during vernalization, AV = after vernalization, SR = shoot apical meristem (reproductive), SV = shoot apical meristem (vegetative), R = replicate.


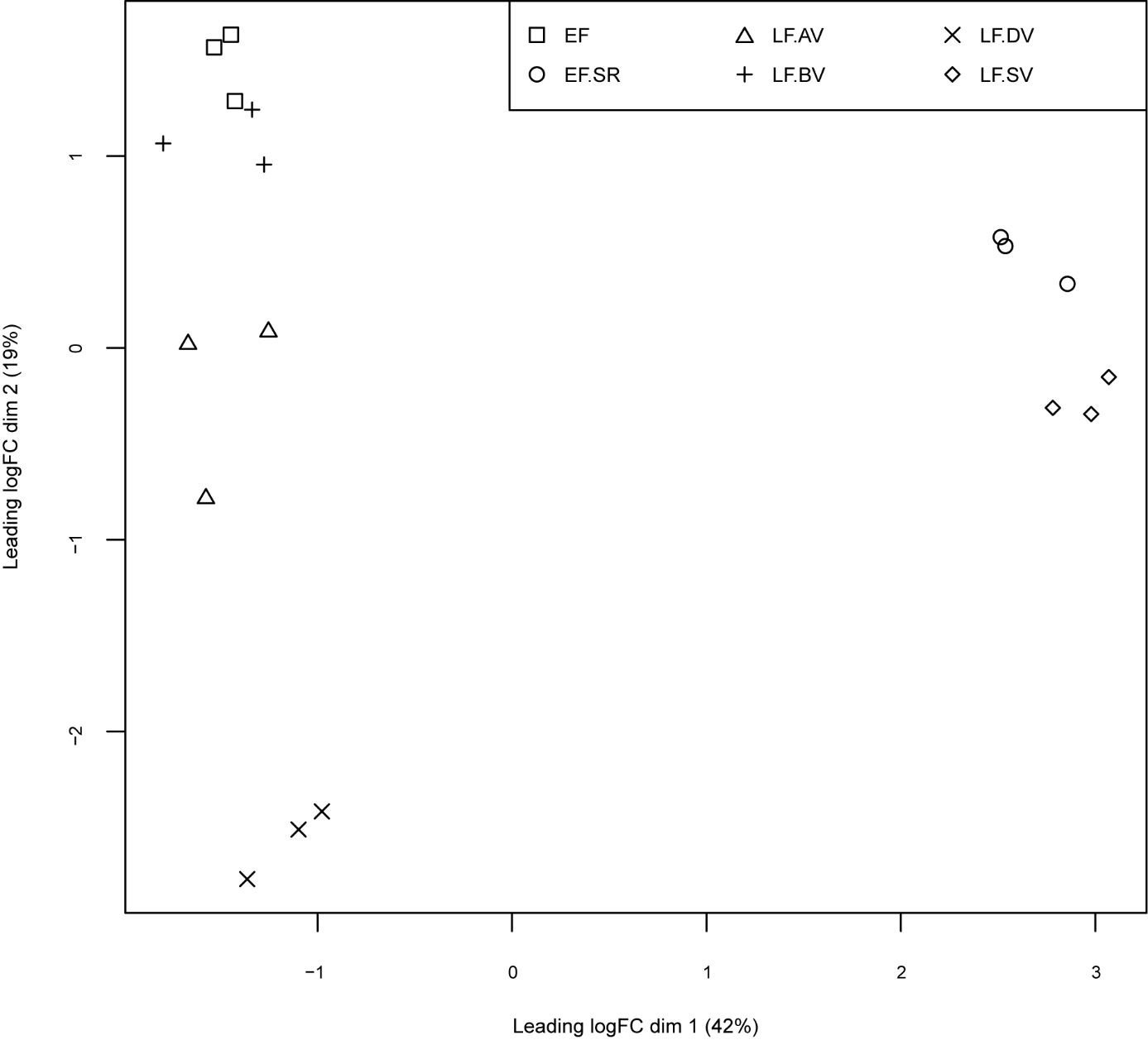


**Figure S4:** Multidimensional scaling plot of each MACE sample.

The plot shows individual replicates (1–3) for each sample. The distance between two points reflects the leading logFC of the corresponding MACE samples, where the leading log­FC is the average of the largest absolute logFC between each pair of samples. EF = early flowering, LF = late flowering, BV = before vernalization, DV = during vernalization, AV = after vernalization, SR = shoot apical meristem (reproductive), SV = shoot apical meristem (vegetative).


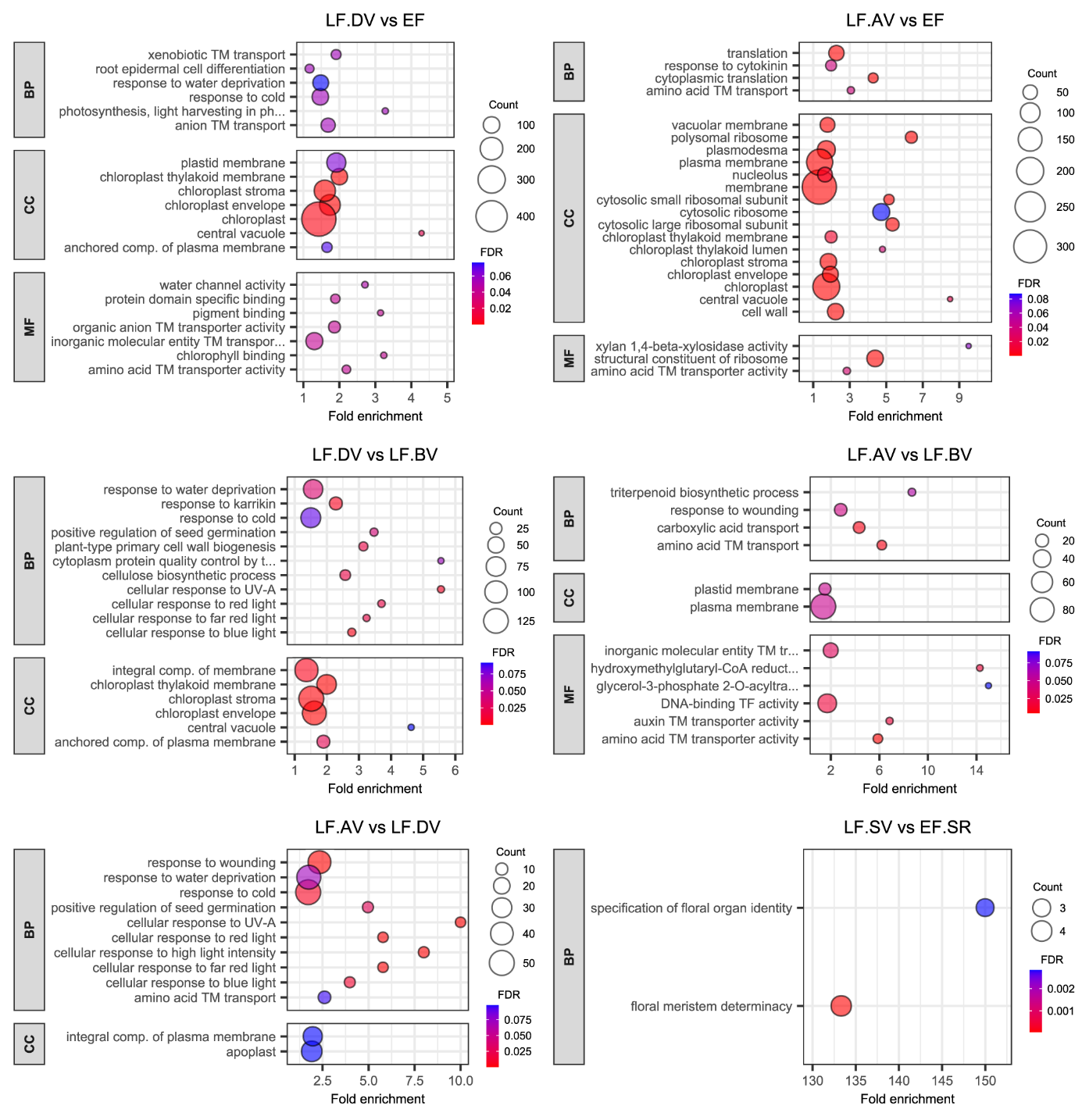


**Figure S5:** Gene Ontology enrichment analysis of differentially expressed genes (DEGs) in the MACE datasets.

Fold enrichment refers to how much more frequently each term was observed than expected. Significance level: FDR < 0.1 (Benjamini-Hochberg). BP = biological process, MF = molecular function, CC = cellular component, EF = early flowering, LF = late flowering, BV = before vernalization, DV = during vernalization, AV = after vernalization, SR = shoot apical meristem (reproductive), SV = shoot apical meristem (vegetative).


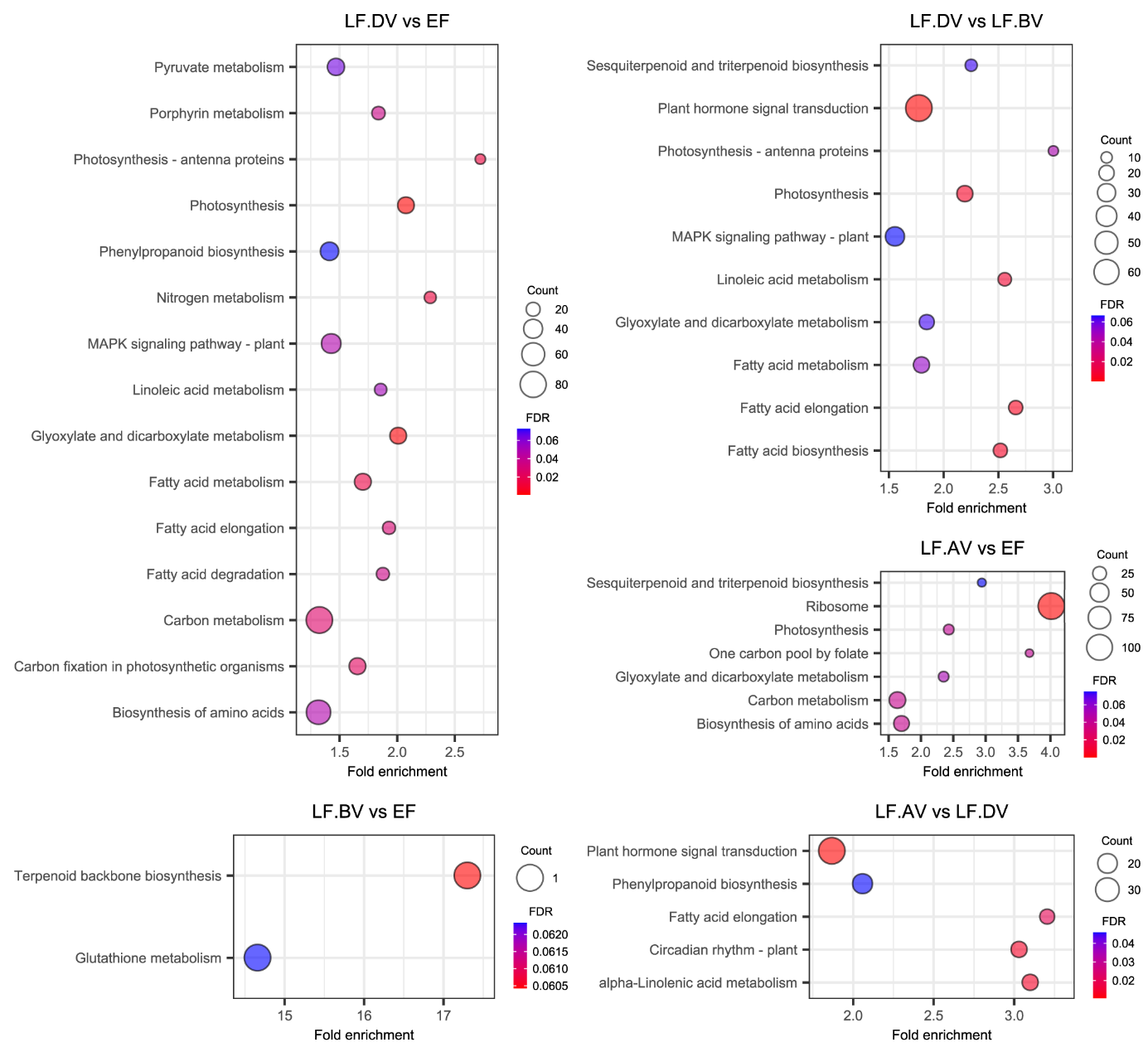


**Figure S6**: KEGG pathway enrichment analysis of DEGs.

Fold enrichment refers to how much more frequently each term was observed than expected. Significance level: FDR < 0.1 (Benjamini-Hochberg). EF = early flowering, LF = late flowering, BV = before vernalization, DV = during vernalization, AV = after vernalization.


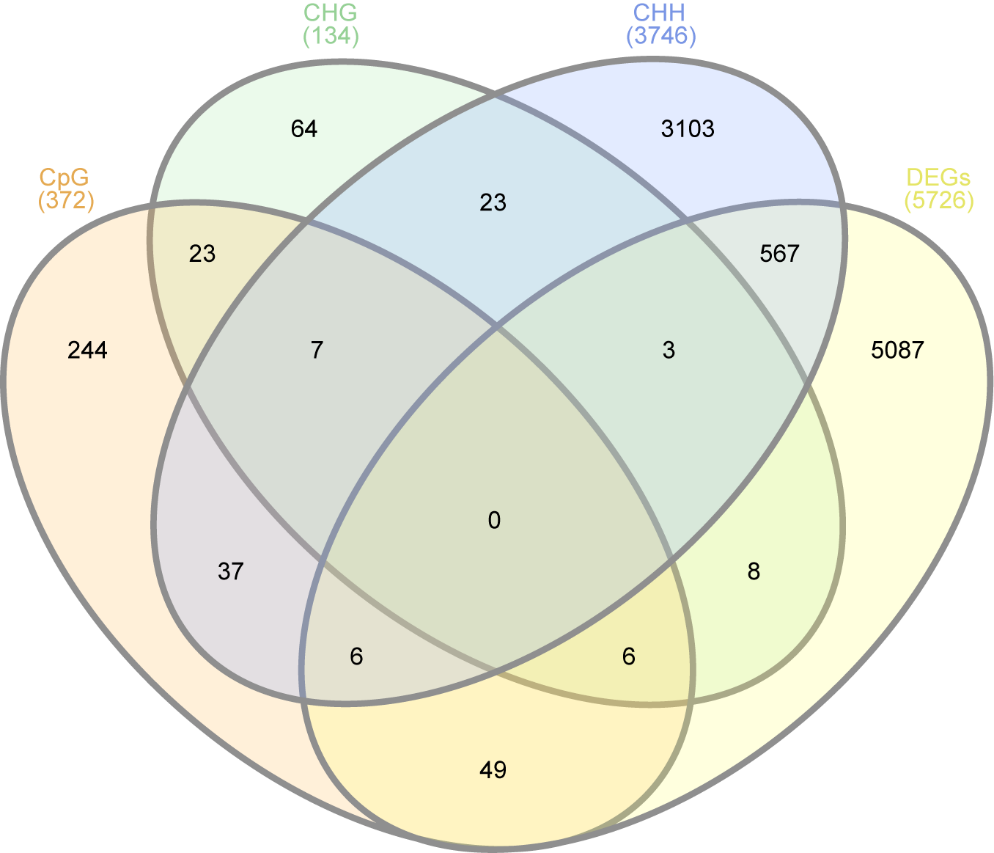


**Figure S7**: Venn diagram of DMR-associated genes (DAGs) in each methylation context and differentially expressed genes (DEGs). DMR = differentially methylated region.


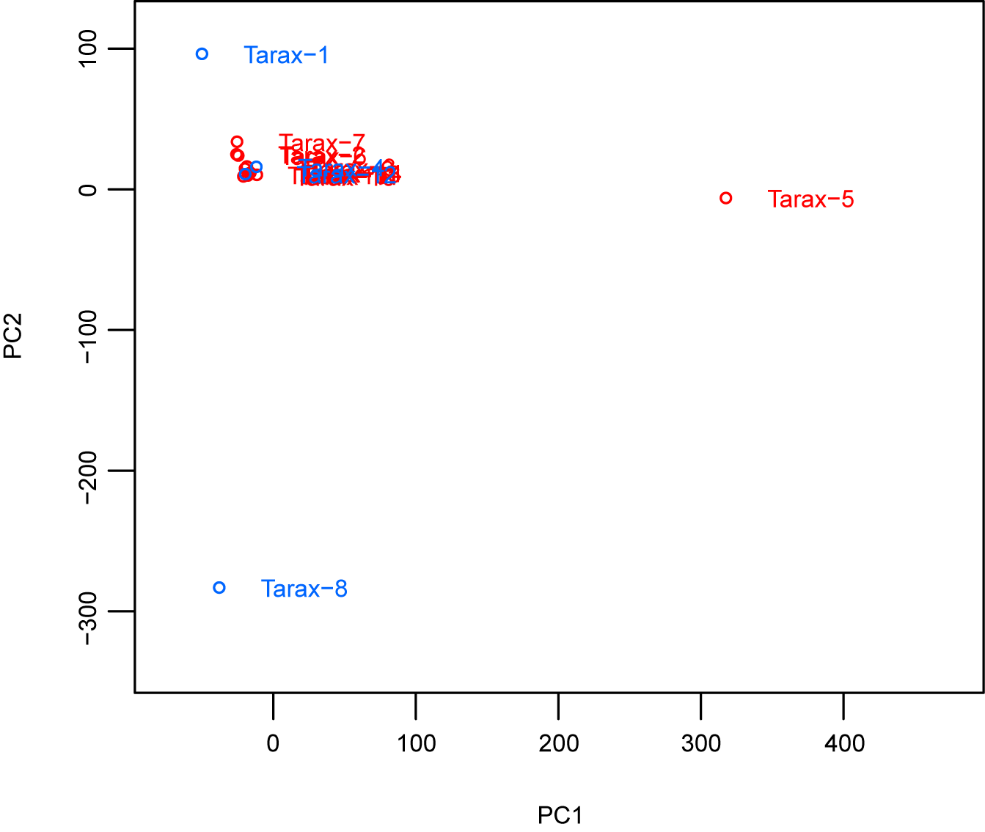


**Figure S8:** Principal component analysis of *T. koksaghyz* samples used for whole genome bisulfite sequencing.

The first two principal components are shown, along with the distribution of samples from early flowering plants in blue and those from late flowering plants in red (Table S1).
